# Diabetes Associated With Greater Ejection Fraction Improvement After Revascularization in Patients With Reduced Ejection Fraction

**DOI:** 10.3389/fcvm.2021.751474

**Published:** 2021-09-27

**Authors:** Shaoping Wang, Bijan J. Borah, Shujuan Cheng, Shiying Li, Ze Zheng, Xiaoyan Gu, Ming Gong, Yi Lyu, Jinghua Liu

**Affiliations:** ^1^Department of Cardiology, Beijing Institute of Heart Lung and Blood Vessel Diseases, Beijing Anzhen Hospital, Capital Medical University, Beijing, China; ^2^Department of Health Sciences Research, Mayo Clinic, Rochester, MN, United States; ^3^Robert D. and Patricia E. Kern Center for Science of Health Care Delivery, Mayo Clinic, Rochester, MN, United States; ^4^Department of Echocardiography, Beijing Institute of Heart Lung and Blood Vessel Diseases, Beijing Anzhen Hospital, Capital Medical University, Beijing, China; ^5^Department of Cardiovascular Surgery, Beijing Institute of Heart Lung and Blood Vessel Diseases, Beijing Anzhen Hospital, Capital Medical University, Beijing, China; ^6^Department of Anesthesiology, Minhang Hospital, Fudan University, Shanghai, China

**Keywords:** diabetes mellitus, ejection fraction, heart failure, revascularization, prognosis

## Abstract

**Objectives:** To investigate the association between diabetes mellitus (DM) and ejection fraction (EF) improvement following revascularization in patients with coronary artery disease (CAD) and left ventricular (LV) dysfunction.

**Background:** Revascularization may improve outcomes of patients with LV dysfunction by improvement of EF. However, the determinants of EF improvement have not yet been investigated comprehensively.

**Method:** A cohort study (No. ChiCTR2100044378) of patient with repeated EF measurements after revascularization was performed. All patients had baseline EF ≤40%. Patients who had EF reassessment 3 months after revascularization were enrolled. Patients were categorized into EF unimproved (absolute increase in EF ≤5%) and improved group (absolute increase in EF >5%).

**Results:** A total of 974 patients were identified. 573 (58.8%) had EF improved. Patients with DM had greater odds of being in the improved group (odds ratio [OR], 1.42; 95% CI, 1.07–1.89; *P* = 0.014). 333 (34.2%) patients with DM had a greater extent of EF improvement after revascularization (10.5 ± 10.4 vs. 8.1 ± 11.2%; *P* = 0.002) compared with non-diabetic patients. The median follow-up time was 3.5 years. DM was associated with higher risk of overall mortality (hazard ratio [HR], 1.46; 95% CI, 1.02–2.08; *P* = 0.037). However, in EF improved group, the risk was similar between diabetic and non-diabetic patients (HR, 1.36; 95% CI, 0.80–2.32; *P* = 0.257).

**Conclusions:** Among patients with reduced EF, DM was associated with greater EF improvement after revascularization. Revascularization in diabetic patients might partially attenuate the impact of DM on adverse outcomes. Our findings imply the indication for revascularization in patients with LV dysfunction who present with DM.

## Introduction

Ischemic etiology is consistently reported as a risk factor for lack of ejection fraction (EF) improvement among patients with heart failure (HF) (1–3). Revascularization including coronary artery bypass grafting (CABG) (4) and percutaneous coronary intervention (PCI) (5–7) may improve long-term outcome by attenuating the ischemic state and reversing left ventricular (LV) remodeling for patients with ischemic HF (8–10). However, the extent and determinants of EF improvement have not been well-investigated (9, 11–14).

The presence of myocardial viability has been shown to be predictive of EF improvement after coronary revascularization (11, 15, 16). However, not all patients with viable myocardium show an improvement of EF. In different studies, about 12% (16) to 64% (11) patients remained EF unimproved after revascularization. Besides myocardial viability, patients with more extensive coronary artery disease (CAD) and worse myocardial dysfunction and remodeling may receive greater benefit from revascularization (17).

Diabetic patients are associated with the decreased utilization of glucose and the increase in myocardial free fatty acids, occurring as a consequence of the mismatch between blood supply and cardiac metabolic requirements (18). These metabolic changes are responsible both for the increased susceptibility of the diabetic heart to myocardial ischemia and for a greater decrease of myocardial performance for a given amount of ischemia, compared to non-diabetic hearts. However, the association between diabetic status and EF improvement following revascularization has not been addressed. We hypothesize that in diabetic patients with LV dysfunction, the effects of revascularization could even give greater benefit than in non-diabetic patients.

Therefore, this study was performed to clarify (1) the determinants of EF improvement after revascularization in patients with preoperative EF≤40%; (2) extent of EF improvement following revascularization in patients with vs. without DM; (3) outcomes difference between diabetic and non-diabetic patients after revascularization in a large clinical cohort with LV dysfunction.

## Materials and Methods

### Patient Selection

This was a real-world retrospective cohort study that used data from Beijing Anzhen Hospital, which is a large referral hospital in China that focuses on heart, lung, and blood vessel diseases. The study was registered in Chinese Clinical Trial Registry (No. ChiCTR2100044378). The study protocol was approved by the hospital's ethics committee.

CAD patients with reduced EF (≤40%) who underwent CABG or PCI with a drug-eluting stent between January 2005 and December 2014, and with repeated EF measurements during follow-up were enrolled. Patients were excluded if they had concomitant non-coronary surgery, were diagnosed as ST-segment elevation myocardial infarction, had only one record of EF follow-up reassessment within 3 months after revascularization. The final study sample included patients who had EF reassessment by echocardiography 3 months after revascularization. Patients were then categorized according to the absolute change in EF: (1) EF unimproved group (absolute increase in EF ≤5%); (2) EF improved group (absolute increase in EF >5%) (19). Patients with EF unimproved were further categorized: (1) EF worsened group (absolute decrease in EF >5%); (2) EF unchanged group (absolute change in EF −5 to 5%) (19).

### Data Collection and Definitions

Baseline demographic, clinical, laboratory, and angiographic parameters for the study patients were ascertained from Beijing Anzhen Hospital medical records. Baseline EF was captured within 30 days before PCI or CABG. Follow-up EF values were defined as the first EF measurement 3 months (20) after revascularization assessed in Beijing Anzhen Hospital. Complete revascularization was defined as successful PCI (residual stenosis of <30%) of all angiographically significant lesions (≥70% diameter stenosis) in 3 coronary arteries and their major branches. A staged procedure within 90 days after discharge was acceptable. For CABG, grafting of every primary coronary artery with ≥70% diameter stenosis was accepted as complete revascularization.

Outcome data were obtained from medical records at Beijing Anzhen Hospital and through telephone follow up. Death was regarded as cardiovascular in origin unless obvious non-cardiovascular causes could be identified. Any death during hospitalization for repeat coronary revascularization was regarded as cardiovascular death. The follow-up time (19, 21, 22) for patients started at the time of the first available EF measurement.

### Statistical Analysis

Only patients with non-missing covariates were included in the study; thus, missing data were not imputed. Continuous variables were expressed as mean (SD) and categorical variables as counts (percentages). Highly skewed continuous distributions were described by median (interquartile range). Baseline characteristics were compared between the EF improved and unimproved groups as well as groups with and without DM by using a student *t*-test, rank sum test, or χ^2^ test, as appropriate. Multivariate logistic regression was used to identify independent correlates of EF improvement. Variables of demographics and history, preoperative echocardiography values, angiography and therapies as well as clinical chemistry were included in the analysis. All variables that had marginal association in univariate analysis (*P* < 0.100) were adopted as independent variables in multivariate logistic regression analysis. Cumulative incidences were estimated by the Kaplan-Meier method and compared by log-rank test. The risks of outcomes were analyzed with a Cox proportional hazards regression model. The proportional hazards assumption was tested for individual covariates and globally on the basis of Schoenfeld residuals. All statistical analyses were based on 2-tailed tests. *P* < 0.05 was considered statistically significant. Statistical analyses were performed with Stata version 14.0 (StataCorp).

## Results

### Baseline Characteristics of EF Improved vs. Unimproved Patients

Among 1,816 initially identified patients, 78 patients who died within 3 months after revascularization, 764 patients were further excluded because EF was not evaluated 3 months after revascularization.

This study cohort included 974 patients who had an initial EF ≤40% and had echocardiography reassessment 3 months after revascularization. The average age at baseline was 64.7 ± 10.9 years (Table 1). Men comprised 83.5% of all subjects. 556 (57.1%) received PCI and 418 (42.9%) underwent CABG. After revascularization, 573 (58.8%) had LVEF improved and 401 (41.2%) remained unimproved. Mean (SD) EF improved significantly, from 35.8% (4.7%) to 52.0% (8.6%), in the EF improved group (*P* < 0.001) and remained reduced (36.9% [3.6%] to 35.4% [5.8%]; *P* < 0.001) in the unimproved group. The mean duration between the preoperative and follow-up EF measurements in two groups was comparable (improved group: 5.9 ± 2.6 months vs. unimproved group: 6.3 ± 2.8 months; *P* = 0.113).

**Table 1:** Patient characteristics at baseline^a^ (EF improved vs. EF unimproved patients).

**Characteristics**	**All patients**	**Improved**	**Unimproved**	***P*-value**
	**(*n* = 974)**	**(*n* = 573)**	**(*n* = 401)**	
**Demographics and history**				
Age, y	64.7 (10.9)	64.8 (11.0)	64.6 (10.7)	0.766
Men	813 (83.5)	475 (82.9)	338 (84.3)	0.565
Weight, kg	72.0 (11.1)	71.7 (11.1)	72.6 (11.0)	0.212
Current smoker	348 (35.7)	206 (36.0)	142 (35.4)	0.863
Hypertension	521 (53.5)	317 (55.3)	204 (50.9)	0.171
eGFR, mL/min/1.73m^2^	85.0 (24.3)	85.4 (23.8)	84.4 (24.9)	0.529
DM	333 (34.2)	216 (37.7)	117 (29.2)	0.006
Insulin-dependent DM	78 (8.1)	46 (8.2)	32 (8.1)	0.951
Cerebral vascular disease	70 (7.2)	37 (6.5)	33 (8.2)	0.292
Atrial fibrillation	45 (4.6)	25 (4.4)	20 (5.0)	0.648
History of MI	452(46.4)	238(41.5)	214 (53.4)	<0.001
History of PCI	176 (18.1)	93 (16.2)	83 (20.7)	0.075
History of CABG	27 (2.8)	14 (2.4)	13 (3.2)	0.455
**Echocardiography**				
**Preoperative**				
EF, %	36.3 (4.3)	35.8 (4.7)	36.9 (3.6)	<0.001
MR (moderate or severe)	166 (17.0)	95 (16.6)	71 (17.7)	0.645
**Postoperative**				
EF, %	45.2 (11.2)	52.0 (8.6)	35.4 (6.2)	<0.001
MR (moderate or severe)	149 (15.3)	55 (9.6)	94 (23.4)	<0.001
Change of EF, %	8.9 (11.0)	16.2 (7.5)	−1.5 (5.4)	<0.001
**Angiography and therapy**				
Multi-vessel disease	769 (79.0)	456 (79.6)	313 (78.1)	0.565
Left main disease	58 (6.0)	35 (6.1)	23 (5.7)	0.809
PCI	556 (57.1)	330 (57.6)	226 (56.4)	0.702
CABG	418 (42.9)	243 (42.4)	175 (43.6)	0.702
Complete revascularization	527 (54.1)	301 (52.5)	226 (56.4)	0.238

*CABG, coronary artery bypass grafting; DM, diabetes mellitus; EF, ejection fraction; eGFR, estimated glomerular filtration rate; MI, myocardial infarction; MR, mitral regurgitation; PCI, percutaneous coronary intervention*.

a *Values are mean (SD) or No. of patients (%)*.

Age at baseline and sex distribution were similar between the EF improved group and EF unimproved group (Table 1). The EF improved group had a significantly higher prevalence of DM (37.7 vs. 29.2%; *P* = 0.006) and lower prevalence of history of myocardial infarction (MI) before revascularization (41.5 vs. 53.4%; *P* < 0.001). The EF improved group had a significantly higher postoperative EF (52.0 ± 8.6 vs. 35.4 ± 6.2%; *P* < 0.001), but lower preoperative EF (35.8 ± 4.7 vs. 36.9 ± 3.6%; *P* < 0.001). The anatomic severity of coronary artery disease was similar between the groups. There was no significant difference in the proportions undergoing revascularization by PCI or CABG, and the groups had similar percentages of complete revascularization.

### Predictors of EF Improvement

Evaluation for independent predictors of EF improvement showed that patients with DM had greater odds of being in the improved group (odds ratio [OR], 1.42; 95% CI, 1.07–1.89; *P* = 0.014) (Table 2). Patients with a history of MI (OR, 0.63; 95% CI, 0.48–0.82; *P* = 0.001) and with higher preoperative EF (OR per 1% increase in EF, 0.94; 95% CI, 0.91–0.97; *P* < 0.001) had lower odds of being in the EF improved group. Neither anatomic severity of coronary vessels (as indicated by multivessel disease and left main disease) nor extent of revascularization (complete vs. incomplete) was an independent correlate of EF improved.

**Table 2:** Baseline factors associated with EF improvement following revascularization in a multivariate model.

**Variables**	**Univariate analysis**		**Multivariate analysis**	
	**OR (95%CI)**	***P*-value**	**OR (95%CI)**	***P*-value**
Age	1.00 (0.99–1.01)	0.766		
Male sex	0.90 (0.64–1.28)	0.565		
Weight	0.99 (0.98–1.00)	0.212		
Current smoker	1.02 (0.78–1.34)	0.863		
Hypertension	1.20 (0.93–1.54)	0.171		
DM	1.47 (1.12–1.93)	0.006	1.42 (1.07–1.89)	0.014
eGFR	1.00 (1.00–1.01)	0.529		
Cerebral vascular disease	0.77 (0.47–1.25)	0.293		
History of MI	0.62 (0.48–0.80)	<0.001	0.63 (0.48–0.82)	0.001
Atrial fibrillation	0.87 (0.48–1.59)	0.648		
History of PCI	0.74 (0.53–1.03)	0.075	0.86 (0.61–1.21)	0.396
Preoperative EF	0.94 (0.91–0.97)	<0.001	0.94 (0.91–0.97)	<0.001
MR (moderate or severe)	0.92 (0.66–1.30)	0.646		
Multivessel disease	1.10 (0.80–1.50)	0.565		
Left main disease	1.07 (0.62–1.84)	0.809		
PCI^*^	1.05 (0.81–1.36)	0.702		
Complete revascularization	0.86 (0.66–1.11)	0.238		

* *CABG was set as reference to PCI*.

### Baseline Characteristics of Diabetic Patients vs. Non-diabetic Patients

To further clarify the characteristics of DM patients, all patients were further categorized according to the status of DM (Table 3). 333 (34.2%) had DM with a mean (SD) hemoglobin A1c of 7.4% (1.5%). The proportions of male patients were lower in diabetic group (79.0 vs. 85.8%; *P* = 0.007). Diabetic patients had a significantly higher prevalence of hypertension (63.7 vs. 48.2%; *P* < 0.001) and cerebral vascular disease (9.9 vs. 5.8%; *P* = 0.018). Diabetic patients had a significantly higher prevalence of multivessel disease (85.3 vs. 75.7%; *P* < 0.001), but lower percentages of complete revascularization (48.4 vs. 57.1%; *P* = 0.009). The proportions undergoing revascularization by PCI or CABG was similar between two groups (*P* = 0.168).

**Table 3:** Patient characteristics at baseline^a^ (diabetic vs. non-diabetic patients).

**Characteristic**	**All patients**	**Diabetic**	**Non-diabetic**	***P*-value**
	**(*N* = 974)**	**(*N* = 333)**	**(*N* = 641)**	
**Demographics and history**				
Age, y	64.7 (10.9)	64.6 (10.2)	64.8 (11.2)	0.766
Men	813 (83.5)	263 (79.0)	550 (85.8)	0.007
Weight, kg	72.0 (11.1)	71.5 (11.2)	72.3 (11.0)	0.325
Current smoker	348 (35.7)	110 (33.0)	238 (37.1)	0.206
Hypertension	521 (53.5)	212 (63.7)	309 (48.2)	<0.001
eGFR, mL/min/1.73m^2^	85.0 (24.3)	85.5 (26.5)	84.8 (23.0)	0.664
HbA1c, %	6.8 (1.4)	7.4 (1.5)	6.1 (1.0)	<0.001
Cerebral vascular disease	70 (7.2)	33 (9.9)	37 (5.8)	0.018
Atrial fibrillation	45 (4.6)	14 (4.2)	31 (4.8)	0.656
History of MI	452 (46.4)	143 (42.9)	309 (48.2)	0.118
History of PCI	176 (18.1)	54 (16.2)	122 (19.0)	0.279
History of CABG	27 (2.8)	15 (4.5)	12 (1.9)	0.018
**Echocardiography**				
**Preoperative**				
EF, %	36.3 (4.3)	36.1 (4.4)	36.4 (4.3)	0.284
MR (moderate or severe)	166 (17.0)	66 (19.8)	100 (15.6)	0.097
**Postoperative**				
EF, %	45.2 (11.2)	46.5 (10.7)	44.5 (11.5)	0.007
MR (moderate or severe)	149 (15.3)	51 (15.3)	98 (15.3)	0.991
Change of EF, %	8.9 (11.0)	10.5 (10.4)	8.1 (11.2)	0.002
**Angiography and therapy**				
Multi-vessel disease	769 (79.0)	284 (85.3)	485 (75.7)	<0.001
Left main disease	58 (6.0)	20 (6.0)	38 (5.9)	0.961
PCI	556 (57.1)	180 (54.1)	376 (58.7)	0.168
CABG	418 (42.9)	153 (46.0)	265 (41.3)	0.168
Complete revascularization	527 (54.1)	161 (48.4)	366 (57.1)	0.009

*CABG, coronary artery bypass grafting; DM, diabetes mellitus; EF, ejection fraction; eGFR, estimated glomerular filtration rate; HbA1c, hemoglobin A1c; MI, myocardial infarction; MR, mitral regurgitation; PCI, percutaneous coronary intervention.*

a *Values are mean (SD) or No. of patients (%)*.

**Figure 1:**
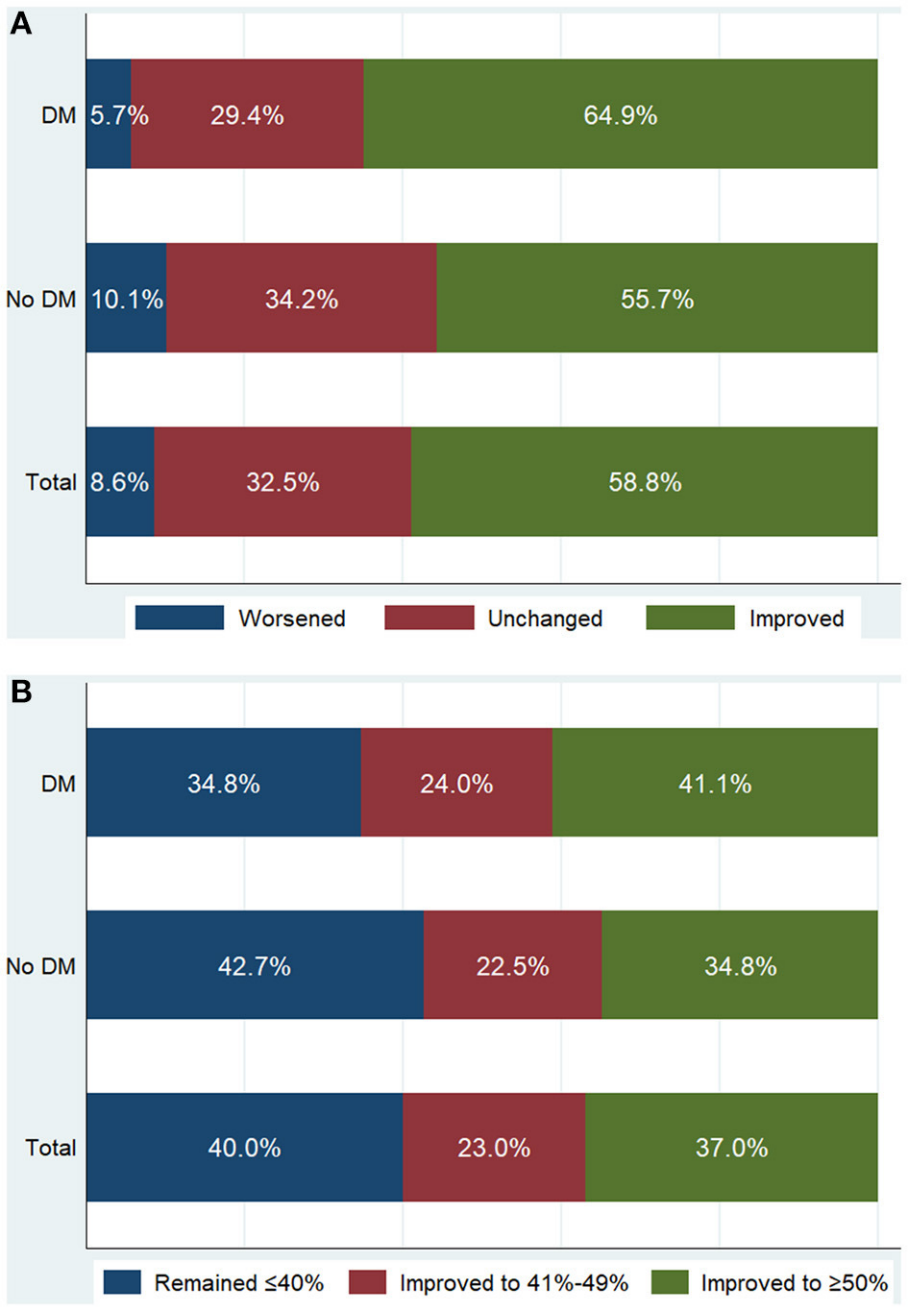
Patient distribution according to the absolute change of ejection fraction (EF) **(A)** and follow-up EF **(B)** after revascularization. Worsened: absolute decrease in EF >5%; Unchanged: absolute change in EF −5% to 5%; Improved: absolute increase in EF >5%. Patients with either worsened EF or unchanged EF were categorized into EF unimproved group. DM, diabetes mellitus.

Compared with non-diabetic patients, diabetic patients had a similar preoperative EF (36.1 ± 4.4 vs. 36.4 ± 4.3%; *P* = 0.284), but had a significantly higher postoperative EF (46.5 ± 10.7 vs. 44.5 ± 11.5%; *P* = 0.007), which resulted in a greater extent of EF improvement after revascularization (10.5 ± 10.4 vs. 8.1 ± 11.2%; *P* = 0.002) (Table 3). After revascularization, in 333 diabetic patients, EF measurements were worsened in 19 (5.7%), unchanged in 98 (29.4%), and improved in 216 (64.9%) (Figure 1A). Diabetic patients were more likely to experience EF improvement compared to non-diabetic patients (*P* = 0.008). After revascularization, 137 (41.1%) diabetic patients had an EF that improved to ≥50%, 80 (24.0%) improved to 41–49%, and 116 (34.8%) remained ≤40% (Figure 1B). Diabetic patients were more likely to have higher EF distribution compared to non-diabetic patients (*P* = 0.049).

### Outcomes of Diabetic Patients vs. Non-diabetic Patients

**Figure 2:**
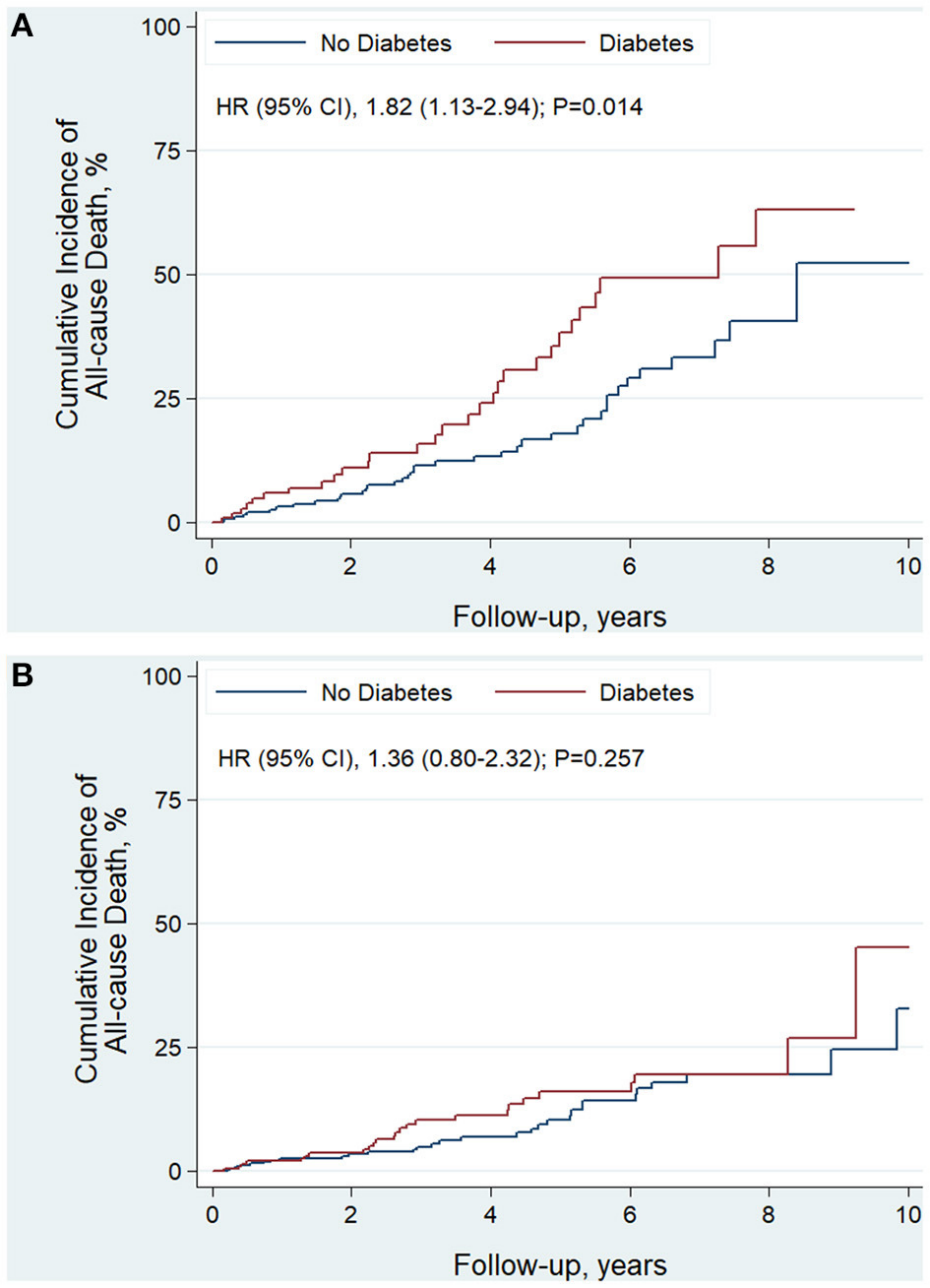
Kaplan-Meier curves estimating incidence of all-cause death after revascularization in EF unimproved group **(A)** and EF improved group **(B)**: diabetic vs. non-diabetic patients.

The median follow-up time was 3.5 years. In EF improved group, there were 55 patients died and 41 were cardiovascular death. In the EF unimproved group, there were 69 patients died and 57 were cardiovascular death. EF improvement was associated with lower risk of all-cause death (HR, 0.45; 95% CI, 0.32–0.64; *P* < 0.001) and cardiovascular death (HR, 0.41; 95% CI, 0.27–0.61; *P* < 0.001). For diabetic status, patient with DM had significantly higher risk of all-cause death (HR, 1.46; 95% CI, 1.02–2.08; *P* = 0.037) and cardiovascular death (HR, 1.48; 95% CI, 1.02–2.22; *P* = 0.046) (Table 4). This finding persisted in EF unimproved group (Table 4 and Figure 2A). However, in EF improved group, both all-cause mortality (HR, 1.36; 95% CI, 0.80–2.32; *P* = 0.257) and cardiovascular mortality (HR, 1.42; 95% CI, 0.77–2.64; *P* = 0.262) were similar between diabetic and non-diabetic patients (Table 4 and Figure 2B).

**Table 4:** Risk of outcomes (diabetic vs. non-diabetic patients).

**Outcomes**	**Total**		**EF improved**		**EF unimproved**	
	**(*****N*** **= 974)**		**(*****N*** **= 573)**		**(*****N*** **= 401)**	
	**HR (95% CI)**	***P-*value**	**HR (95% CI)**	***P*-value**	**HR (95% CI)**	***P*-value**
**All-cause death**						
DM	1.46 (1.02–2.08)	0.037	1.36 (0.80–2.32)	0.257	1.82 (1.13–2.94)	0.014
No DM	Ref		Ref		Ref	
**Cardiovascular death**						
DM	1.48 (1.02–2.22)	0.046	1.42 (0.77–2.64)	0.262	1.82 (1.07–3.08)	0.026
No DM	Ref		Ref		Ref	

*DM, diabetes mellitus; EF, ejection fraction; HR, hazard ratio; Ref, reference*.

## Discussion

In the current study, EF measurements 3 months after revascularization were used to define the patient cohorts with EF improved or EF unimproved. The literature has not addressed the issue of EF evolution over time after revascularization (12, 14). Three months after revascularization might be necessary allowing for the LV to recover from CABG surgery or PCI procedure and for ischemic myocardium to fully reverse remodeling after revascularization and medical therapy (20). According this definition, about 60% CAD patients with reduced EF (≤40%) had LVEF improved (absolute increase in EF >5%) following revascularization by either PCI or CABG, which indicated the benefit of revascularization in patients with LV dysfunction. Patients with EF improved had significantly lower risk of overall and cardiovascular death compared to those with EF unimproved.

In the current study, without history of MI, lower preoperative EF and DM were identified as three independent predictors of EF improvement after revascularization among patients with LV dysfunction. Patients who had history of MI are more likely to have scarred and non-viable myocardium compared to patients without history of MI. Thus, patients with history of MI might benefit less from revascularization and had lower odds to have EF improved. The multicenter Surgical Treatment for Ischemic Heart Failure (STICH) trial (23) compared the efficacy of medical therapy alone with that of medial therapy plus CABG in patients with LV dysfunction (EF ≤35%). Preoperative EF <27% was one of three prognostic factors which associated with greater survival benefit from CABG (17). This result consisted with current finding and supported the indication for revascularization in patients with worse myocardial dysfunction.

Besides history of MI and preoperative EF, more important finding in this study is that DM was firstly identified, to our knowledge, as a factor associated with greater EF improvement after revascularization. Patients with DM have an impaired myocardial metabolism and increased cardiac cell death with its attendant fibrosis, which causing an accelerated and diffused atherosclerosis (18). Clinical studies in the scenario of ACS have shown evidences of a higher susceptibility of diabetic patients to such things as greater risk for the development of heart failure and higher morbidity and mortality rate (24–26). However, by treatments targeting those pathogenetic factors caused by DM, diabetic patients may benefit more than non-diabetic patients. In diabetic patients with ischemic LV dysfunction, modulation of free fatty acids metabolism could give greater benefits of decreasing the incidence of angina attacks than in non-diabetic patients (27). The addition of direct renin inhibitor aliskiren to standard therapy was associated with trends toward greater reduction in LV size among diabetic compared with non-diabetic subjects who had history of MI and EF ≤45% (28). An invasive strategy appeared to reduce recurrent non-fatal MI to a greater extent in patients with DM and non-ST-segment elevation acute coronary syndromes (29). In current study, DM was found as an independent correlate of greater EF improvement among patients with LV dysfunction who underwent revascularization. Revascularization is a direct strategy to reverse the mismatch between blood supply and cardiac metabolic requirements in ischemic heart. This mismatch was more severe in diabetic compared with non-diabetic myocardium, which therefore may result in greater improvement of EF by revascularization. In our patient cohort, DM was associated with greater EF improvement. In EF improved group, diabetic patients had similar risk of overall and cardiovascular mortality compared with non-diabetic patients. In contrast, in EF unimproved group, DM was associated with higher risk of both overall and cardiovascular death. This indicated that greater EF improvement by revascularization in diabetic patients at least partially attenuated the impact of DM on adverse outcomes.

Completeness of revascularization achieved with CABG is associated with a bigger number of treated lesions and a more percentage of successful complete revascularization when compared to a PCI strategy. A large pooled analysis of over 5,000 diabetic patients with stable CAD demonstrated the benefit of completeness of revascularization and long-term patency of CABG, which provides protection from subsequent re-infarction likely contributing to the survival advantage (30). In the current study, despite the high prevalence of multivessel diseases, the incidence of CABG tended to be lower than that of PCI in both diabetic and non-diabetic group. Diabetic patients had a significantly higher prevalence of multivessel disease, but lower percentages of complete revascularization. Extent of revascularization (complete vs. incomplete) was not an independent correlate of EF improvement. Whether the severity of LV dysfunction and/or extent of coronary disease would affect the revascularization strategic decision-making, and the benefit of complete revascularization needs to be further clarified.

This was a non-randomized, observational study from a single center. Therefore, as with any observational study, ours also suffered from selection biases. In this study, only EF measurements that were obtained by echocardiography in one hospital were used for comparison, since institutions and specific methods for measuring EF vary. This restriction improved the accuracy of the EF measurements but increased the number of excluded patients. The median follow-up time of the study was 3.5 years. Taking into account the natural slow evolution of the disease and its related complications, relative short time period of follow-up is another limitation. In addition, we highlighted that the impact of DM on outcomes in this study was actually evaluated among patients who are survivors 3 months at least after PCI or CABG because each patients had EF reassessment at least 3 months after revascularization.

## Conclusion

The current study indicates that ~60% of patients with preoperative EF ≤40% are likely to have absolute EF improved (>5%) after revascularization. DM was an independent predictor of EF improvement and associated with greater EF improvement. Revascularization in diabetic patients might partially attenuate the impact of DM on adverse outcomes. Our findings imply the indication for revascularization in patients with LV dysfunction who present with DM.

## Data Availability Statement

The raw data supporting the conclusions of this article will be made available by the authors, without undue reservation.

## Ethics Statement

The studies involving human participants were reviewed and approved by Ethics Committee of Beijing Anzhen Hospital. Written informed consent for participation was not required for this study in accordance with the national legislation and the institutional requirements.

## Author Contributions

JL and YL conceived the concept of the study and supervision. SW contributed to the design of the research and wrote the original-draft. All authors were involved in data collection and analysis, edited, and approved the final version of the manuscript.

## Conflict of Interest

The authors declare that the research was conducted in the absence of any commercial or financial relationships that could be construed as a potential conflict of interest.

## Publisher's Note

All claims expressed in this article are solely those of the authors and do not necessarily represent those of their affiliated organizations, or those of the publisher, the editors and the reviewers. Any product that may be evaluated in this article, or claim that may be made by its manufacturer, is not guaranteed or endorsed by the publisher.

## Abbreviations

CABG: coronary artery bypass grafting
CAD: coronary artery disease
DM: diabetes mellitus
EF: ejection fraction
eGFR: estimated glomerular filtration rate
HbA1c: hemoglobin A1c
HF: heart failure
HR: hazard ratio
LV: left ventricular
MI: myocardial infarction
MR: mitral regurgitation
OR: odds ratio
PCI: percutaneous coronary intervention.
